# Flipped classroom frameworks improve efficacy in undergraduate practical courses – a quasi-randomized pilot study in otorhinolaryngology

**DOI:** 10.1186/s12909-018-1398-5

**Published:** 2018-12-04

**Authors:** Tobias Dombrowski, Christian Wrobel, Stefan Dazert, Stefan Volkenstein

**Affiliations:** Department of Otorhinolaryngology, Head and Neck Surgery, Ruhr University Bochum, St. Elisabeth-Hospital, Bleichstr. 15, 44787 Bochum, Germany

**Keywords:** Medical education, Flipped classroom, Otorhinolaryngology, Practical course, Undergraduate

## Abstract

**Background:**

Curriculum design and specific topic selection for on-site practical courses in clinical disciplines with limited teaching time is challenging. An electronic learning supported curriculum based on the flipped classroom principle has a high potential to effectively gain knowledge and education along with improving practical experience. Here, we demonstrate the introduction of a flipped classroom curriculum for practical courses in Otorhinolaryngology (ORL) in real world practice to improve the on-site time management and students’ experience.

**Methods:**

Educational aims of our practical curriculum were analysed and rearranged into a flipped classroom (FC) framework. Core knowledge was taught preliminary based on a moodle platform in predominantly interactive formats. Two quasi-randomized groups were formed with 212 participants either receiving or not receiving access to the e-learning program to reduce a potential allocation bias to the e-learning group. All students completed a questionnaire with learning related items. Focusing the study on the intervention group, we investigated if students using the flipped classroom more often felt better prepared for the practical course.

**Results:**

The online learning platform was highly accepted and frequently used by 66% of participating students in the e-learning group. Students with frequent use of our e-learning platform significantly felt better prepared for the practical course (*p* = 0.001). The far majority of all students supports the idea of further development of e-learning. More than 70% were generally interested in ORL. Handouts were the overall most important learning resource and more than 50% relied solely on them.

**Conclusions:**

Flipped classroom curricula can save time and help improving the on-site experience in practical courses especially in smaller surgical disciplines. The acceptance of digital learning is high, and most students rely on handouts for learning ORL, emphasizing the need for guidance by the teacher e.g. through electronic learning. Our results underline the high potential of FC to address teaching challenges for smaller medical disciplines with limited teaching time like ORL.

**Electronic supplementary material:**

The online version of this article (10.1186/s12909-018-1398-5) contains supplementary material, which is available to authorized users.

## Background

While electronic learning (e-learning) approaches have become popular in teaching, face-to-face learning environments are still most commonly used in undergraduate medical education. Although offering a broad range of useful supplements to classical teaching, the existing medical e-learning resources are poorly integrated into framework curricula [1]. Therefore, there is still a lot of potential for reasonably combining e-learning and face-to-face teaching into structured learning environments [2]. Regarding undergraduate Otorhinolaryngology (ORL) teaching, we even see particular significance for e-learning applications. First, classical learning strategies guided by textbooks and lecture notes are strongly influenced by educational resources available online, potentially increasing unguided and unstructured learning. Second, within undergraduate medical education curricula worldwide, ORL topics are usually considered low priority and have limited teaching hours leading to short exposure periods to this subject [2–5]. However, the core topics of ORL and the practical skills involved are of high importance for numerous other disciplines in medicine, e.g. general medicine or pediatrics [2]. Students are therefore individually responsible to bridge these divergences which often results in poor preparation prior to practical courses and consequentially reduced benefit [4, 5]. Furthermore, by including the special equipment and examinations that are required in ORL clinics, only a small portion of students feel prepared for common clinical ORL conditions [6].

Recently, the concept of the flipped classroom was proposed as a modern and suitable model for medical education and has been established mainly for learning preclinical subjects like anatomy or biochemistry. Students autonomously study both the basic and theoretical aspects of a given topic. Subsequently, they meet with the lecturer to gain deeper insights and to work through problem-solving exercises [1, 7–9]. Supported by recent reviews and a meta-analysis about flipped classrooms in medical education, this teaching concept may have a high potential for improving knowledge and education also in ORL, along with improving practical experiences [10–12]. Therefore, based on Prober and Khan’s suggestions for introducing structured e-learning programs and the flipped-classed principle into undergraduate medical education, we have developed a novel framework curriculum based on the development of an online platform that runs in conjunction with our practical ORL course [1, 13]. We expected a relevant effect on our practical course due to reduced on-site theoretical introduction and more participation in clinical workflows, optimizing the on-site ORL experience of students and teachers. Here, we present the innovative design of our learning environment, along with an evaluation of the students’ experience.

## Methods

Changing the curriculum of our practical ORL course was primarily driven by the idea to improve student’s clinical experience in ORL. While suffering from limited teaching time (2 days, 5 h each) during this mandatory course for all medical students, teachers felt that students usually were poorly prepared and the practical experience was significantly reduced by the introduction to basic ORL topics. Searching for potential improvements without increasing the predetermined on-site teaching time, we felt that the flipped classroom principle may be useful to approach this problem. The flipped classroom is based on the idea, that students autonomously prepare basic aspects of a topic, the ‘theory’, before meeting the teacher for deeper insights and problem-solving exercises, the ‘homework’ [1, 7–9]. Thus, the classical roles of students and teachers, as well as the chronology of a course or lecture are ‘flipped’.

To develop our flipped-classroom’s structure, we followed the suggestions of Prober and Khan (2013) [1].

**Fig. 1:**
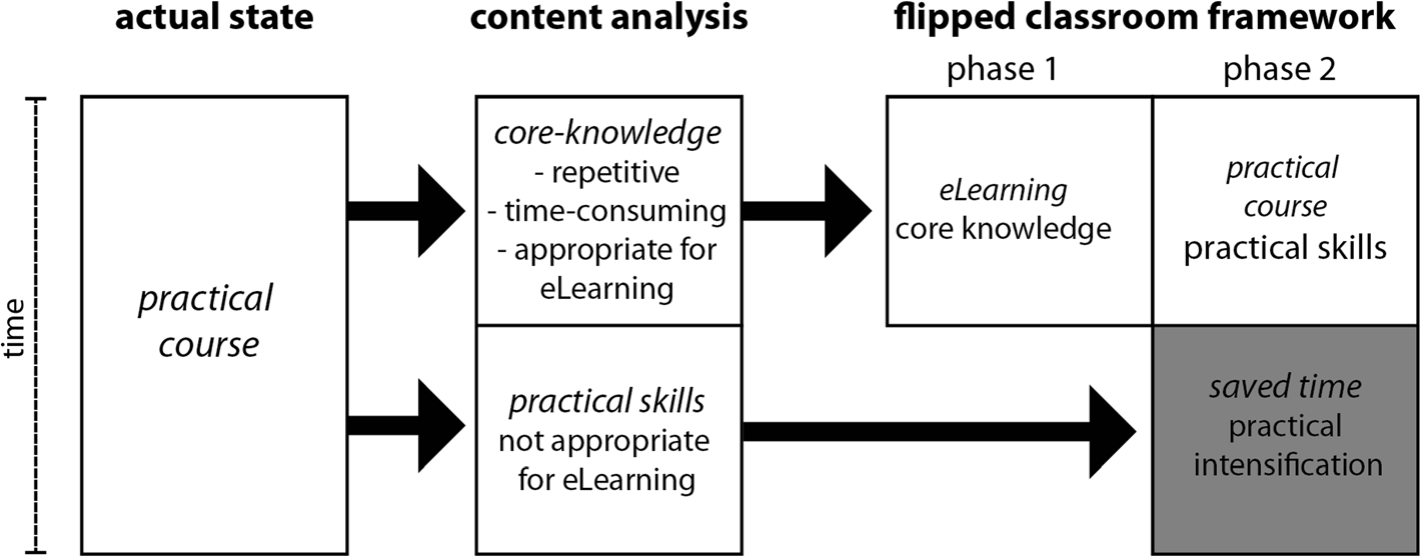
Structured workflow for the development of a flipped classroom framework for ENT practical courses. After identifying the key content and topics of the practical course, the core-knowledge can be studied at home (phase 1), while phase 2 represents the on-site practical course. The saved time can then be used for practical intensification

First, we examined our practical curriculum and identified repetitively taught time-consuming content that was deemed appropriate for online teaching (Fig. 1). Afterwards, we separated educational aims in this respect. Hence, we defined the “framework of core knowledge” [1] and the educational objectives for the practical course (Table 1).

**Table 1:** Educational aims of online vs. practical courses

Item	Online course – gaining knowledge	Practical course – applying knowledge
Anatomy & physiology	• Refreshing knowledge	• Identifying anatomical landmarks • 3D orientation
Medical history	• Special features of ORL- anamnesis	• Perform ORL-adapted anamnesis
ORL-examination	• Examination structure • Instruments - Names - Purpose - Handling – theoretically • Physiological findings	• Reproducing structured examination • Practice handling of instruments • Identifying non-physiological findings
Further diagnostics	• Basics - Audiometry - Vestibular diagnostics - ENT-radiology	• Recognizing pathological audiometry/vestibular diagnostics • Indication for radiological examinations
Surgery	• Principles of common surgeries • Behavioural rules (operating room)	• Better orientation when visiting the operating room

Secondly, we implemented our learning environment based on the open source Moodle learning management system (LMS). We produced videos that were shorter than five minutes each containing information about the clinical examination, e.g. of the ear, rhinoscopy and endoscopy along with a presentation of anatomical landmarks. Other relevant topics were taught in predominantly interactive formats (e.g. guided clinical cases, quizzes on visual diagnoses or tests). For those interested in deeper insights, we proposed supplemental material (e.g. principles of ORL surgeries) and advanced non-mandatory courses corresponding to Prober ad Khan’s step three.

To investigate the students’ success (does the flipped classroom improve the on-site ORL experience?) and satisfaction (is the flipped classroom appropriate?) with the flipped classroom curriculum, we planned an evaluation based on a questionnaire (Additional file 1). The study was accompanying the introduction of the flipped classroom curriculum into the mandatory, 2 × 5 h ORL practical course in the fifth year of medical studies, equivalent to the third clinical year. Students were assigned to the practical course by the university’s medical education office based on scheduling purposes and evenly distributed over a whole semester without influence by the authors. Therefore, launching the online platform and new curriculum at midterm created two quasi-randomized, single-blinded groups with participants either receiving (after midterm) or not receiving access (before midterm) to the e-learning program. The main purpose of this randomization was to prevent a selection of e-Learning-attracted students in the intervention group (allocation bias). Both groups completed a questionnaire immediately following their practical course containing thirteen e-learning or learning related items. The study focused on the questionnaire for the e-learning group in order to assess the benefit of the e-learning platform for the practical course, as comparing a group with a massive learning intervention to a control without is usually not meaningful [14]. The group without e-learning was anyway asked about their ORL-related learning behaviour, their general interest in ORL and their opinion about e-learning. As the main idea behind the flipped classroom framework was to improve student’s preparation for the practical course and their ORL experience (success), the primary outcome in the intervention group was to analyse, if students using the moodle platform more frequently felt better prepared for and gained knowledge during the practical course compared to less intensive users. Those questions were based on a four-point Likert scale (strongly agree – agree – disagree – strongly disagree). A general evaluation of the moodle course on a six-point Likert scale (1 = very good, 6 = very bad, equivalent to german usual grading system) was also included to assess satisfaction with the technical implementation of the flipped classroom.

Besides the questionnaires and the above-mentioned groups, we analysed user statistics of our e-learning platform for three years after its launch based on the internal moodle statistics module.

According to our local ethics committee’s guidelines, a formal application was not necessary for our study. However, neither the collection of demographic data nor a comparison with results of the general ORL-assessment at the end of the semester was permitted.

The statistical analysis was performed with R Software for statistical computing (The R Foundation). *P*-values were considered significant below 5% threshold. Analyzed data was tested for normality with the Shapiro-Wilk test. Depending on dataset and question, we used Wilcoxon rank sum test, two-sided proportional test or Fisher’s exact test to test for significance.

## Results

The study included 212 participants, resulting in group sizes of 103 students without and 109 students with access to the e-learning program. The internal consistency was acceptable for both types of questionnaires (Cronbach’s α = 0.74 without, α = 0.78 with additional e-learning items).

**Fig. 2:**
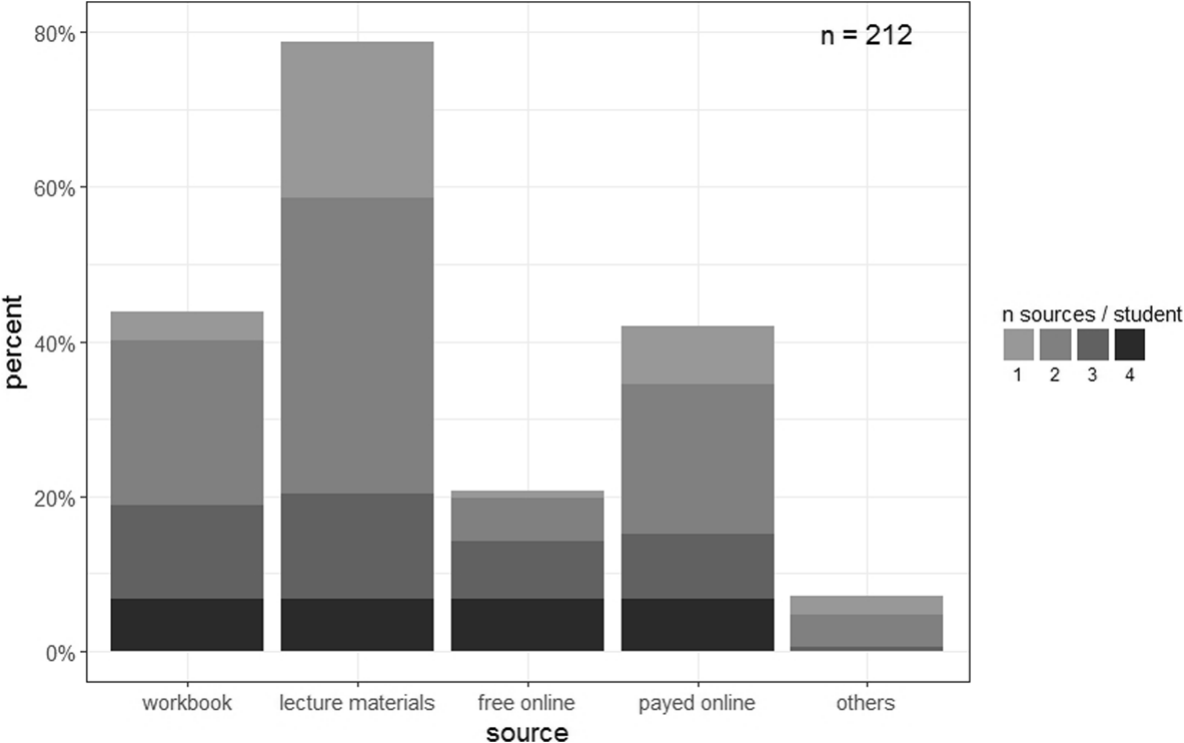
Relative frequency of learning resources in relation to the total number of participants. The stacked barplots also display the total number of learning resources used by participating students. The e-learning and non e-learning group were summarized to clarify the presentation

For both study groups, we first asked about the use of educational resources to study ORL courses. Textbooks were used by half or less of the students (38.9% of the non-e-learning group vs. 51.4% of the e-learning group), whereas handouts tended to be highly important in both groups (69.9% vs. 92.2%, respectively). Students in the e-learning group used “free online resources” more often (10.7% vs. 51.5%, respectively). There were also students using costly online course materials (33% vs. 53.4%, respectively). A minority of students (7.8% vs. 6.8%, respectively) used other resources. The e-learning students in this study tended to use more than one learning resource (1 vs 2 median, *p* < 0.05 Wilcoxon signed-rank test). Among students using a single source only for learning (50.4% of the non-e-learning group vs 14.7% of the e-learning group), 57.7 and 56.3% respectively relied solely on handouts. A summary of both groups’ results and their count of learning resources is displayed in Fig. 2.

**Fig. 3:**
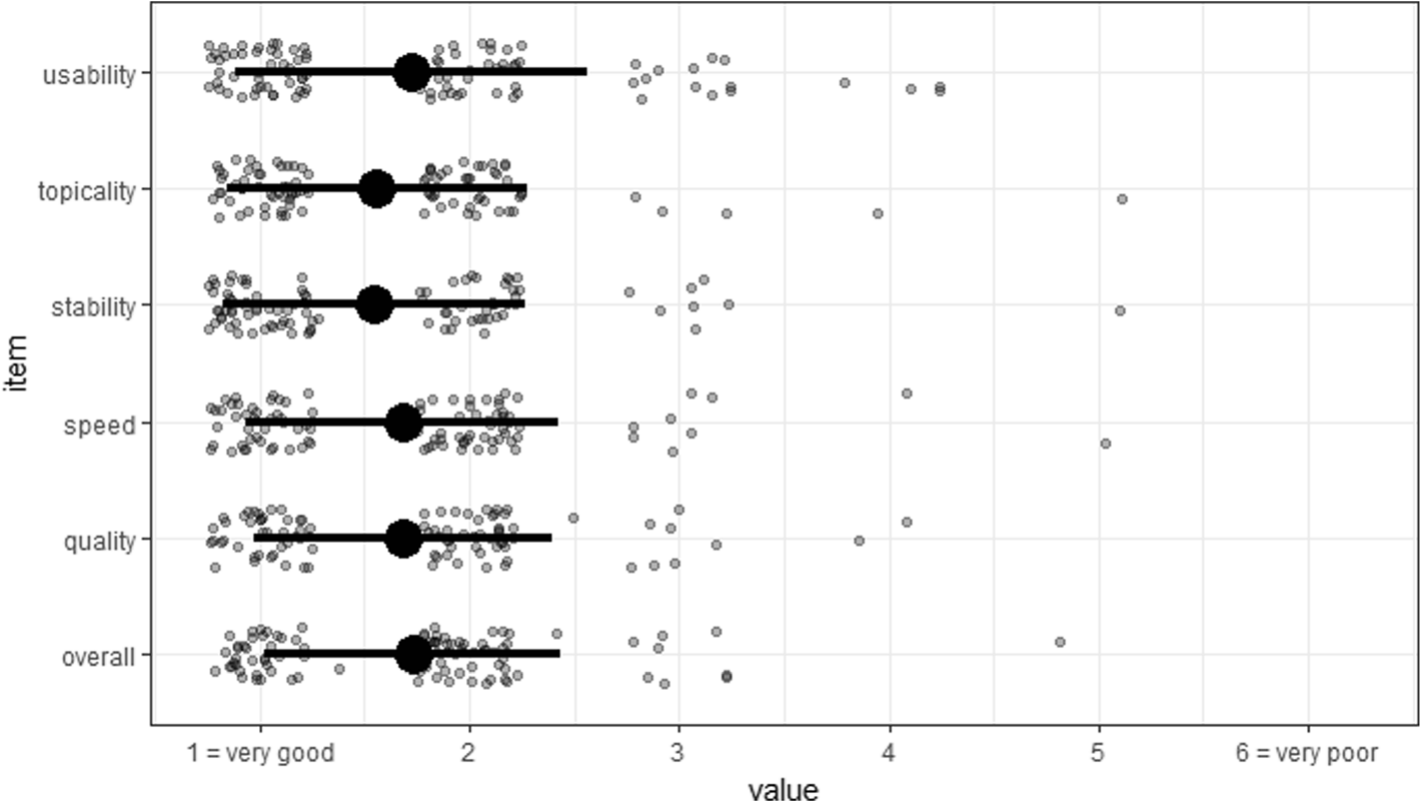
Summarized individual ratings for each of the shown items on the e-learning course (scale: 1 = very good, 6 = poor, whisker = SD, black dot = mean)

A general evaluation of our Moodle e-learning environment was provided by the e-learning participants (Fig. 3). Altogether, a very good evaluation of stability, speed, ease of use, quality, and topicality was consistent with average scores between 1.54–1.75 recorded for all items.

Within the e-learning group, students were asked how well prepared they felt for the practical course, if our e-learning course itself prepared them sufficiently for the practical component and if they gained knowledge after participating in our flipped classroom curriculum (see Table 2). Here, we excluded the data for the analysis, if one or more of those questions were incompletely answered. The majority agreed or strongly agreed to all of those questions. A small number disagreed and no one strongly disagreed. The results were divided into subgroups by the frequency of using the e-learning for preparation (*frequently* = went through the preparation items completely at least once, *occasionally =* went through some parts of the preparation completely, *rarely* = did not complete any content). Students using the e-learning program a lot tended to more often strongly agreed to all three items on the questionnaire. The difference was statistically significant for the item “E-learning prepared sufficiently”, *p* = 0.001, Fisher’s exact test.

**Table 2:** Overview of the items “I feel well prepared”, “e-learning prepared sufficiently”, “I gained knowledge” related to the frequency of using the e-learning course

Question (*n* = 62 each)		Strongly agree	Agree	Disagree	Strongly disagree	N/A
I generally feel well prepared		14 (22.6%)	37 (59.7%)	4 (6.5%)	0 (0%)	7 (11.3%)
Use of e-learning	frequently	13 (21.0%)	25 (40.3%)	0	0	3 (4.8%)
	occasionally	1 (1.6%)	10 (16.1%)	1 (1.6%)	0	2 (3.2%)
	rarely	0	2 (3.2%)	3 (4.8%	0	2
E-learning prepared myself sufficiently^a^		14 (22.6%)	38 (61.3%)	1 (1.6%)	0 (0%)	9 (14.5%)
Use of e-learning	frequently	13 (21.0%)	24 (38.7%)	1 (1.6%)	0	3 (4.8%)
	occasionally	1 (1.6%)	11 (17.7%)	0	0	2 (3.2%)
	rarely	0	3 (4.8%)	0	0	4 (6.5%)
I gained knowledge		18 (29.3%)	31 (50.0%)	5 (8.1%)	0 (0%)	8 (12.9%)
Use of e-learning	frequently	10 (16.1%)	24 (38.7%)	4 (6.5%)	0	3 (4.8%)
	occasionally	5 (8.1%)	6 (9.7%)	1 (1.6%)	0	2 (3.2%)
	rarely	3 (4.8%)	1 (1.6%)	0	0	3 (4.8%)

^a^significant difference between subgroups (frequency vs Likert), Fisher’s exact test

In both study groups, we asked for students’ general interest in ORL and in further development of e-learning on a 4-point Likert scale to investigate, if participation in the flipped classroom may improve both. Here, 79.2% of the non-e-learning group vs. 72.9% of the e-learning group agreed to be strongly interested (21.8% vs. 22.4%) or interested (57.4% vs. 50.5%) in ORL. Only 18.8% vs. 25.2% stated to have a low interest while 2% vs. 1.9% said to have no interest in ORL (not significant, Fisher’s exact test). Regarding participants’ opinions on whether further development of e-learning programs would be meaningful, less students in the “non-e-learning” group strongly agreed (41.9% non-e-learning vs. 53% e-learning group), with more agreeing overall (46.6% vs. 40.2%, respectively). Only 8.1% vs. 6.9% disagreed, and 3.5% vs. 0% strongly disagreed, respectively (not significant, Fisher’s exact test).

In addition to the above-mentioned questionnaires, we analyzed the user statistics of our e-learning platform for three years after its launch indepently from the study. Median click rates per month increased from 184.5 (minimum 0, maximum 605 clicks per month) in the first year to 1203 (127–9886 clicks/month) in the third year. Additionally, 66.1% (*n* = 62) reported using the e-learning program “frequently”, while 22.6 and 11.3% did this “occasionally” or “rarely”. The most frequently used category on the e-learning program was “learning with clinical cases” (average: 1674 clicks/year), while “examination videos” had the lowest click rates (average: 145 clicks/year). Maximum click rates were always registered shortly before written ORL exams, lowest during semester breaks.

## Discussion

Here, we presented the first flipped classroom design for undergraduate practical courses in otorhinolaryngology and its evaluation by medical students. With 212 participants, resulting in group sizes of 103 students without and 109 with e-learning experience, we were able to include a reasonable number of medical students.

Within the general evaluation, an overall high satisfaction with the Moodle LMS corresponds to its wide acceptance in higher education nowadays along with its open source availability and simplicity to customise. Looking at consistently high satisfaction rates for quality and content as well as a high overall rating our content appears appropriate for its purpose. The analysis of average click rates for our core videos explaining ORL examination approximately correspond to the number of participating students. While we were neither allowed nor technically able to link this data with personal learning behaviour, we could speculate that most students watched these videos only once. If so, this could be an indication that eight videos of approximately 2 min are near the reasonable amount of time for preparation. But this aspect is hard to assess and still a matter of discussion among medical education professionals [15]. The popularity of our category “learning with clinical cases” can be explained with their similarity to the current design of written exams in Germany and their capability to virtually rebuild clinical decision strategies.

Increasing medical applications of flipped classrooms were published recently but mostly in the context of preclinical courses e.g. anatomy or biochemistry [7, 8]. Regarding clinical undergraduate education, Lin et al. compared a flipped classroom with lecture-based teaching for two theoretical lessons in ophthalmology a discipline with similar demands as ORL regarding teaching time and relevance among students. Both teachers and students were more satisfied with the flipped classroom design compared to lecture-based learning and students had better experiences of problem solving, creative thinking and team working [16]. After consequently implementing a flipped classroom in our practical course curriculum, we experienced a noticeable benefit as introduction time could be reduced to a minimum allowing an immediate start with practical teaching contents. This observation corresponds well to our finding that students using e-learning frequently felt to be prepared better. Those students also significantly more often agreed to have gained knowledge suggesting a positive effect of the flipped classroom design on the on-site learning experience. In our case, only one student disagreed that our flipped classroom prepares well for the practical course. Similar findings were described in other studies of the implementation of flipped classroom formats in undergraduate medical education [12, 16–18]. We agree that at least parts of these results are due to increased teaching and learning activities like Cheng et al. proposed recently [8].

Another interesting result of our study indicates a change of paradigm concerning the resources for learning ORL. Online resources have become more popular in the recent years, since < 50% of the students in this study using textbooks for learning. Issued lecture materials or handouts were most frequently used suggesting a still existing orientation towards classical learning and teaching concepts. This fact emphasizes the necessity of providing a suitable curriculum since approximately 30% of the students in the group without access to the e-learning program relied solely on materials issued for studying ORL. As students in the e-learning group used significantly more resources for learning ORL in parallel most of them used our voluntary e-learning platform as an additional tool to their normal learning strategies.

Our data do not support our initial thesis that medical students may be more interested in studying ORL courses if effective teaching concepts are provided. Anyway, we showed that a general interest in ORL was expressed in almost two thirds of all our participants. Furthermore, despite the use of the e-learning platform being voluntary two thirds of the students reported using it frequently for preparation of the practical course and around 90% of all students desired further development of our e-learning concept. We conclude, that the initially observed poor preparation for our practical courses triggering the curriculum change, does not seem to be a consequence of poor general interest in ORL topics. Anyway, our flipped classroom based strategy may help motivating students to pass a distinct and focused preparation before the course.

Our proposed model for a flipped classroom design is a suitable strategy to compete against students’ poor experience of studying ORL in undergraduate medical education. Consistent with existing literature, we showed that our concept generated high satisfaction rates and students reporting the impression of being better prepared when using the e-learning program. Therefore, we agree with other educational experts’ in the opinion that the flipped classroom is a valuable concept that can be applied supporting general teaching methods in medical education. One could speculate, that our model could also be translated to other medical discplines with limitied teaching time besides ORL.

Designed as a proof of concept study in real world practice, our work has several limitations. While we could demonstrate the feasibility of our e-learning approach, a control group showing the superiority of the flipped classroom to other approaches is missing. This is a result of both integrating the study into the defined environment of a mandatory practical course, as well as the complex and time-consuming design of a control group, that is also exposed to increased teaching and learning activities. Additionally, the study is impaired by the lack of validated assessment tools, especially for practical skills in ORL. Our results therefore demand for external validation and follow-up research, e.g. including investigation of students’ participation in clinical workflows, satisfaction of teachers and an individual assessment of practical skills. The latter part would require a personalized data collection and assessment, which was in our case not approved by the local ethics committee.

## Conclusions

The flipped classroom is an appropriate model for designing medical e-learning courses that support teaching and learning activities. Saved time for introducing new topics can improve the on-site ORL practical experience making it more effective and attractive for students. This could add a benefit for teaching curricula in smaller clinical disciplines as frequent e-learning users felt better prepared for practical courses in our study. Therefore, we added evidence that flipped classroom based curricula have a potential to fulfil not just ORL-related teaching challenges.

## Additional file

Additional file 1: Questionnaires. (DOCX 14 kb)

## Abbreviations

e-learning: electronic learning
FC: Flipped classroom
ORL: Otorhinolaryngology
