# Supplementary material for: Flipped classroom frameworks improve efficacy in undergraduate practical courses – a quasi-randomized pilot study in otorhinolaryngology
Source: BMC Med Educ. 2018 Dec 4;18:294. doi: 10.1186/s12909-018-1398-5 (PMC6280380; doi:10.1186/s12909-018-1398-5)
Supplement: Supplementary file 1 — Questionnaires. (DOCX 14 kb) [file 12909_2018_1398_MOESM1_ESM.docx]

**Questionnaire e-Learning group**

1. Which educational resource do you usually use for learning Otorhinolaryngology ?

Internet (paid)

Internet (free)

workbook

issued (lecture) materials / handouts

others

1. Generally I am interested in ENT topics

Strongly agree agree disagree strongly disagree I don’t know

1. Did you feel prepared for the practical course?

Strongly agree agree disagree strongly disagree I don’t know

1. I gained knowledge during the practical course

Strongly agree agree disagree strongly disagree I don’t know

1. I would appreciate further development of e-Learning

Strongly agree agree disagree strongly disagree I don’t know

1. The e-Learning courses prepare well for the practical course

Strongly agree agree disagree strongly disagree I don’t know

1. I used the moodle course

A lot average little (or not at all)

Please rate the following aspects of our e-Learning platform (1= very good, 6=very bad)

1. Stability ___
2. speed ___
3. ease of use ___
4. quality of content ___
5. design ___
6. topicality of content ___
7. overall rating ___

**Questionnaire without e-learning items**

1. Which educational resource do you usually use for learning Otorhinolaryngology ?

Internet (paid)

Internet (free)

workbook

issued (lecture) materials / handouts

1. Generally I am interested in ENT topics

Strongly agree agree disagree strongly disagree I don’t know

1. I would appreciate further development of e-Learning

Strongly agree agree disagree strongly disagree I don’t know
